# Engineering of Semiconductor Nanocrystals for Light Emitting Applications

**DOI:** 10.3390/ma9080672

**Published:** 2016-08-09

**Authors:** Francesco Todescato, Ilaria Fortunati, Alessandro Minotto, Raffaella Signorini, Jacek J. Jasieniak, Renato Bozio

**Affiliations:** 1 Department of Chemical Science and U.R. INSTM, University of Padova, Via Marzolo 1, Padova I-35131, Italy; ftodescato@yahoo.it (F.T.); ilaria.fortunati@unipd.it (I.F.); minotto.ale@gmail.com (A.M.); raffaella.signorini@unipd.it (R.S.); 2 Department of Materials Science and Engineering, Monash Energy Materials and Systems Institute (MEMSI), Monash University, 22 Alliance Lane, Room 109, Clayton 3800, Australia; Jacek.Jasieniak@monash.edu

**Keywords:** quantum dots, display, photoluminescence, laser emission, electroluminescence, LED

## Abstract

Semiconductor nanocrystals are rapidly spreading into the display and lighting markets. Compared with liquid crystal and organic LED displays, nanocrystalline quantum dots (QDs) provide highly saturated colors, wide color gamut, resolution, rapid response time, optical efficiency, durability and low cost. This remarkable progress has been made possible by the rapid advances in the synthesis of colloidal QDs and by the progress in understanding the intriguing new physics exhibited by these nanoparticles. In this review, we provide support to the idea that suitably engineered core/graded-shell QDs exhibit exceptionally favorable optical properties, photoluminescence and optical gain, while keeping the synthesis facile and producing QDs well suited for light emitting applications. Solid-state laser emitters can greatly profit from QDs as efficient gain materials. Progress towards fabricating low threshold, solution processed DFB lasers that are optically pumped using one- and two-photon absorption is reviewed. In the field of display technologies, the exploitation of the exceptional photoluminescence properties of QDs for LCD backlighting has already advanced to commercial levels. The next big challenge is to develop the electroluminescence properties of QD to a similar state. We present an overview of QLED devices and of the great perspectives for next generation display and lighting technologies.

## 1. Introduction

Liquid crystal displays (LCD) and organic light emitting diodes (OLED) are the two major technologies competing within the $100 bn display market [1], each with its own advantages and disadvantages. LCD is leading in lifetime, power consumption, resolution density and cost; comparable to OLED in ambient contrast ratio and viewing angle, but inferior in such fundamental requirements as color and brightness, module thickness/flexibility and response time [1,2,3].

The recent implementation of nanocrystalline quantum dots (QDs) for the backlighting of LCD displays has provided a competitive advantage over OLED. Thanks to their intrinsic optical properties, such as their broad absorption band but narrow emission spectra, high fluorescence quantum yield, high photostability and controllable emission and surface properties [4,5,6], QD based down-conversion displays are now showing performances comparable or even better than OLED devices. QDs enable highly saturated colors, a wider color gamut and a comparable response time, while retaining advantages in cost, resolution, optical efficiency and durability. For display applications, QDs can be used either exploiting their photoluminescence for LCD backlight unit or their electroluminescence for QD-light emitting diodes (QLED). In fact, advances in this display technology are expected from the development of QD-based light emitting diodes (QLED) currently underway [7,8].

This remarkable progress has been made possible by the rapid advances in the synthesis of colloidal QDs and by the progress in understanding the intriguing new physics exhibited by these nanoparticles and how it relates to their structure [9,10]. In the last few decades wet synthesis strategies have achieved an enormous progress in producing a great variety of colloidal nanostructures with controlled chemical and physical properties. Indeed, semiconductor nanocrystals (NCs), consisting of between 100 and 10,000 atoms, can now be readily synthesized with unprecedented control of size (1–10 nanometers), distribution (<10% polydispersity), shape (spherical, elongated, branched, hollow, etc.) and composition (e.g., IV, II-VI, I-III-VI, I-II-IV-VI grouped material). Of all the underlying phenomena that are demonstrated at the nanoscale, the quantum size effect is arguably the most important. It occurs when the physical dimensions of a NC become smaller than the characteristic lengths of the quantum states of charge carriers (electrons and holes) in the ground or in their excited states—a condition that is known as quantum confinement. The Coulomb attraction between oppositely charged carriers drives a spatial localization of bound electron-hole pairs, i.e., excitons. This phenomenon makes the optical absorption and emission properties of a given material size-dependent, with band peaks shifting to shorter wavelengths at smaller sizes [11,12,13,14,15,16].

It is well known that the optical absorption and emission properties of semiconductor quantum dots (QDs) depend on an intricate interplay of factors, beside their chemical composition (whether elemental, II-VI, III-V, etc.), their dimensions and shape. The composition and chemistry of their surfaces and the defect distribution, whether at surfaces or interfaces, is of paramount importance. Furthermore, for practical applications QDs are used as ensembles and suffer from inherent polydispersity factors. Unraveling this complexity to gain a complete understanding of the physical, optical and electrical properties of QDs has remained a difficult research challenge.

However, through progressive improvement in QD quality, largely enabled by a better understanding of the mechanisms for their synthesis and of the fundamental physical processes underlying their properties, significant progress has been made in achieving nearly ideal photophysical properties. Recent advances in nanostructure assembly have combined the spatial confinement of elementary excitations with the impressive design flexibility provided by self-assembly into 2D or 3D superstructures [17,18]. A set of inorganic components has been identified as distinct building blocks in order to design and fabricate specific systems suited for engineering energy transfer (ET) [19] and charge transfer (CT) [20] processes through systematic control on dimensionality, rate and flow direction in structures characterized by the combination of zero-dimensional systems, (e.g., quantum dots), one dimensional systems (wires, tubes and rods) and 2D systems (platelets) in solution. Multilayer nanostructures for QLED applications are indeed hybrid multifunctional systems in which QD layers are sandwiched between inorganic and/or organic layers for electron and hole transport [21].

For all these reasons, it has been possible to use QDs in lucrative applications ranging from biological labels [22], solar cells [23] and light emitting devices [24,25,26]. For such applications, QDs possess sufficiently strong light absorption properties and/or efficient and stable emission to be considered superior compared to molecular organic dyes. These features, including one- and two-photon absorption, are direct consequences of the quantum confinement effect and of the atom-like density-of-states of excitons at moderate energies above the bulk energy gap of the constituent semiconductor material. All the oscillator strength for optical transitions is concentrated at discrete energies that are strongly dependent on the size and shape of the QD. In this review, we will briefly investigate the QD physical features that can mainly affect the optical properties, in particular to increase their emission efficiency. In the second part, we will report some results regarding their practical use as active materials for optical gain applications and as light emitters in QLED and display devices.

## 2. Core/Shell Quantum Dots: What a Suitably Designed Shell Can Do

In the most simplified picture, the electron (*e*) and hole (*h*) forming the exciton are just two oppositely charged particles trapped in the same nanometric volume [11]. The wavefunction distribution of both electrons and holes determines the strength of the Coulomb forces binding the *e-h* pair. An efficient confinement is expected to enhance the overlap of wavefunctions, thus making the radiative decay rate very fast. This, combined with a reduced non-radiative decay due to exciton-phonon coupling [27], leads one to expect fluorescence quantum yield (QY) values approaching unity.

This ideal situation should be contrasted with the more complicated structural features and dynamical processes of a real QD. Roughly speaking, the deviation from ideal behavior can be traced back to two main categories of phenomena: (i) the presence of a large number of defects at the surface and/or within the QDs, which cause trapping of either electrons, holes, or both; and (ii) the activation of a very efficient non-radiative decay mechanism known as *Auger recombination (AR*) by the aforementioned charged defects or due to the presence of more than one exciton in a single QD [11].

Surfaces present a major fundamental challenge for QDs because, depending on their size, ~10%–80% of all their atoms are located at the surface, where they remain only partially coordinated. These unsaturated surface dangling bonds act as efficient charge traps that drastically lower the QY [16].

The first strategy developed to saturate these dangling bonds is through organic passivation. In this process, a suitable organic ligand works as a capping agent for the surface atoms, while also providing the QD with solubility in a given solvent. Typical ligands include trioctylphospine (TOP), trioctylphospine oxide (TOPO), oleic-acid (OA) and various aliphatic amines (e.g., oleylamine, octylamine, etc.) [9,16]. Through the use of such passivants, the low QY of unpassivated QDs (commonly <1%) can be partially increased to typical values of between 1% and 50%. However, in addition to providing limited chemical stability, one of the major caveats of organic passivation stems from steric hindrance, which reduces the surface packing density to below that required for complete surface coordination of the dots [28].

**Figure 1:**
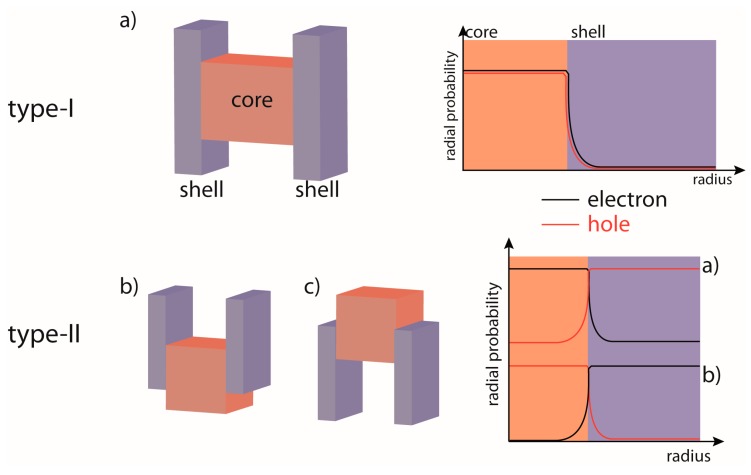
Schematic representation of band alignment and carriers distribution in Type-I (**a**) and Type-II QDs (**b**,**c**).

A more elegant solution that overcomes both of these limitations relies on the epitaxial growth of an inorganic shell layer around the core. A specific choice of core and shell materials may determine different electronic configurations depending on how the conduction band (CB) and valence band (VB) edges of bulk materials line up in the shell with respect to the core. In the so-called Type-I configuration the band edges of the shell encompass those of the core (see Figure 1a). This type of band alignment leads to the formation of potential barriers that effectively contain the extent of the *e* an *h* wavefunctions to within the core volume and away from the surface [11,13,14,15,29]. This property is also confirmed by the plot of the *e* and *h* radial probabilities reported in Figure 1a-right panel. Using Type-I core/shell QDs (for example: CdSe/CdS, CdSe/ZnS, InAs/CdSe), QYs up to unity have been achieved [30]. Compositional engineering of the core/shell structure can also favor preferential charge separation through a Type-II configuration (for example: CdTe/CdSe, CdSe/ZnTe, InP/CdS) [31,32,33] (see Figure 1b,c). Such a structure provides control of the spatial overlap between electrons and holes; thus, enabling a facile way to manipulate Coulomb interactions in nanostructures. This is important for developing efficient light emitting devices, non-linear optical applications and photovoltaics [29].

### Trends towards Type-II QDs for Energy Applications

In contrast to Type-I nanocrystals, Type-II QDs exhibit strongly red-shifted emission (up to ~150 nm) originating from the radiative recombination of electron and hole across the core-shell interface, which would otherwise be impossible to achieve from either only core or only shell Type-I materials, or from *giant* QDs [34,35,36]. The emission wavelength can be tuned from 700 to 1000 nm by modifying the shell thickness and the core size. This large Stokes shift strongly reduces the self-reabsorption of emission, a process that generally worsens the conventional “phosphor” materials performances, giving new opportunities for the production of new generation light emitting devices [36]. In addition, among different shaped Type-II nanostructures, the fluorescence lifetimes of the CdSe/CdTe core/crown nanoplatelets are measured to be two orders of magnitude greater (up to 190 ns) than those of the core-only Type-I nanoplatelets, thanks to the presence of the spatially indirect excitons at the Type-II interface [34,37].

Considering also the electronic properties of such nanostructures, quasi Type-II and Type-II QDs are highly promising materials as light harvesting materials for photovoltaic [38,39,40] and photoconduction applications, where efficient removal of excited charges is required. It has been demonstrated that heterostructures show higher photon to current conversion efficiency with respect to single-component QD, thanks to the ability to tailor the band offsets by selectively modifying the size of one constituent [32,33,41]. The poor stability, the difficult embedding in a high quality film of such nanostructures (in particular asymmetric ones) and the demanding physical charge extraction from the core, are the main challenging issues to be addressed to increase their large use in practical devices [42].

## 3. Synthesis Strategy and Structural Characterization

### 3.1. Nanocrystal Synthesis Strategies

The precise control of the electron and hole spatial distributions within QDs through tailored inorganic core/shell heterostructures, provides the required tuning of optical, electronic and chemical properties to be suitable for a wide range of potential applications. The origin of core/shell structures in colloidal systems stems from the pioneering work of Henglein and Brus in the 1980s. In particular, Henglein showed that the chemisorption of inorganic ions on semiconductor crystallites has drastic influences on the resulting photoluminescence (PL) properties [43]. Brus made use of this factor to be the first in reporting the growth of a ZnS monolayer (ML) around a CdSe core [44]. The low temperatures utilized in such syntheses gave rise to low QY (<1%), due to the defective core, core/shell interface and poor passivation of the exterior surface dangling bonds. Following the advent of the hot-injection method in 1993 by Murray and co-workers to grow high quality II-VI QD cores [45], in 1996 Hines and Guyot-Sionnest used organometallic precursors to grow a 0.6 nm ZnS shell on high quality CdSe colloidal QDs. They achieved unprecedented QYs of up to 50% [46]. In close succession, detailed studies of CdSe/ZnS and CdSe/CdS growth were reported by the groups of Bawendi [47] and Alivisatos [48], highlighting the influence of shell composition and thickness on the extent of carrier delocalization and postulating on the importance of interfacial core/shell lattice strain.

**Figure 2:**
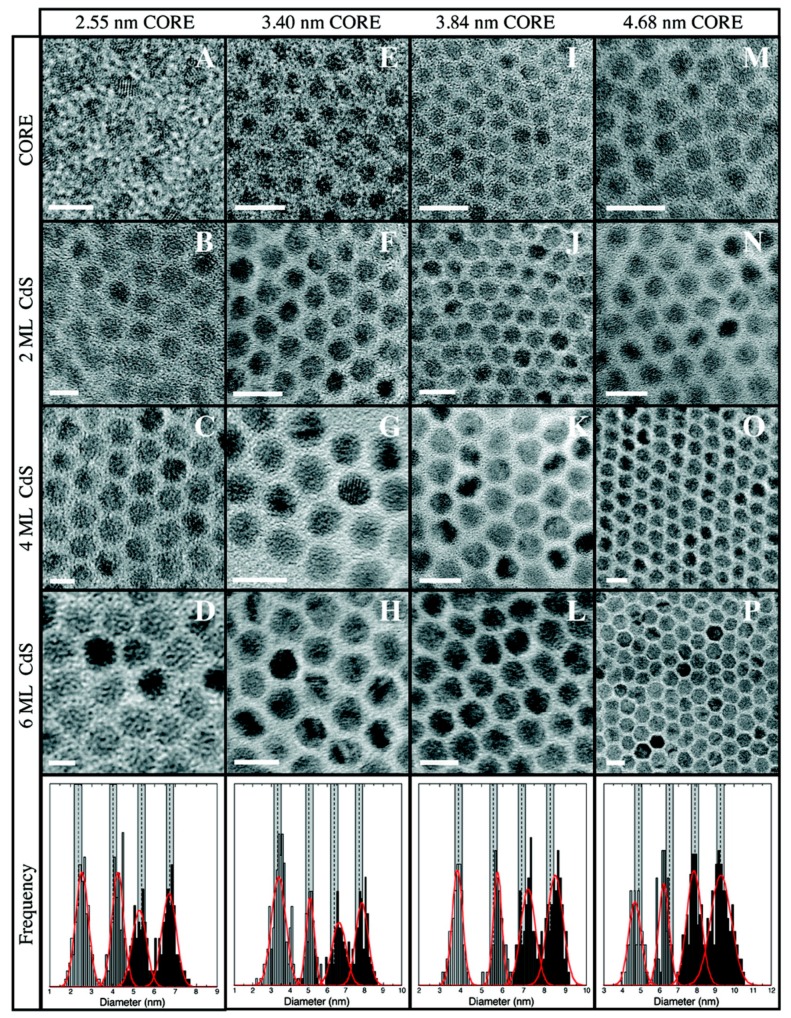
High-resolution transmission electron micrographs of CdSe cores of varying size overcoated by 2, 4 and 6 MLs of CdS. Scale bar equals 5 nm for Panels **A**–**D** and 10 nm for Panels **E**–**P**. Histograms of the measured particle sizes are included for reference, with the dashed lines indicating the predicted size based on the quantity of monomer added. Reprinted with permission from reference [50]. Copyright 2009 American Chemical Society.

Of all the synthetic methods used for the growth of core/shell QDs, the successive ion layer adsorption and reaction (SILAR) protocol developed by Li et al. has been the most versatile [49]. Borrowing from the concepts used in molecular beam epitaxy, SILAR relies on the separate introduction and growth of individual shell precursors monolayer by monolayer. As demonstrated for CdSe/CdS in Figure 2, this control has enabled highly precise QD heterostructures to be grown in a predictable manner [50]. Moreover, its versatility has been exploited for the synthesis of a wide variety of core/shell heterostructures, including CdSe/CdS/ZnS graded QDs [51], ultra-thick shell CdSe/CdS QDs (up to 20 ML shell) [52], CdS/CdSe/CdS QD-quantum wells [53], and CdTe/CdSe Type-II QDs [54].

**Figure 3:**
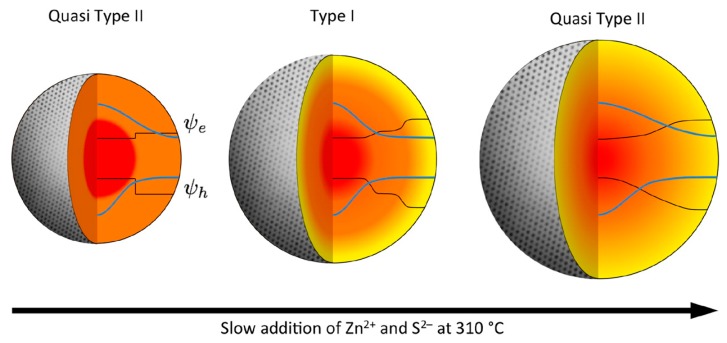
Electronic structure of core/shell/shell QD during ZnS shell growth and alloying. The shell initially confines both e and h to the core (center), until extended alloying smoothes out the potential well and the electron wavefunction spreads out over the whole structure in a quasi Type-II configuration (right). Reprinted with permission from reference [57]. Copyright 2013 American Chemical Society.

Since SILAR procedures are generally time-consuming and complex, other one-pot methods have been developed. Microwave-assisted syntheses of CdSe/CdS/CdZnS core-shell-shell QDs with high luminescence and excellent stability in aqueous solutions [55] and of Cd based nanocrystals [56] have been tested. This method presents some advantages with respect to injection-based syntheses, such as the selective activation of the target precursor, the high reproducibility from batch to batch, the near-continuous nanocrystal production. Another one-pot synthesis for the epitaxial growth of a graded CdS/ZnS shell onto CdSe core has been reported, giving QDs with superior QY (above 90%) due to the strong and controlled alloying between two shells which was promoted by the treatment at 310 °C (see Figure 3) [57].

During the last decade, also microfluidics platforms have been improved for the large-scale production of nanocrystals with good control on physical and chemical properties. This improvement has been made possible thanks to the increased inertness of reactors, the integration of sensors for the real-time analysis during each process step and the optimization of algorithms to increase the volume production [58,59]. Nanocrystalline colloids of wide range of materials have been synthetized in microreactors, such as CdTe, CdSe [60], InP [61], or even CdSe/ZnS and ZnSe/ZnS core/shell structures [62]. Albeit the microfluidic approach could in future substitute the batch syntheses, additional improvements are required for the synthesis of particles with more complex compositions and shapes and with controlled photophysical properties.

Recently, a thermal cycling with single-source precursors method has been optimized for the growth of CdSe/CdS core-shell QDs protected with thiol ligands, by means of a ligand exchange process with alkanethiol at room temperature. The obtained nanocrystals possess high crystallinity and QY, thanks to the surface defects passivation with thiols [63].

### 3.2. Structural Characterization of Core-Shell Nanocrystals

At the heart of core/shell QDs is the interface that forms between the core and the shell during growth: the features characterizing the interface region strongly define the final optical properties of the quantum dot [64]. Standard Raman spectroscopy and its surface-enhanced (SERS) analogue have emerged as powerful experimental techniques to probe such interfaces, because they provide direct signatures of the bulk and surface Raman modes from the various materials within a core/shell heterostructure [65]. By comparing the evolution of such bulk and surface modes, SERS has clearly identified that for CdSe-based core/shell structures interfacial alloying is present for CdS shells up to a thickness of 1 nm (i.e., ~3 ML), while it is limited to only 0.3 nm (i.e., ~1 ML) for Cd_0.5_Zn_0.5_S [66,67]. This finding is consistent with the reduced lattice strain expected between CdSe and CdS (~4%) compared to Cd_0.5_Zn_0.5_S (~8%). Dabbousi’s early work on CdSe/ZnS, which has a large lattice mismatch of ~12%, clearly showed that the core/shell interfacial integrity is maintained only up to ~1–2 MLs, before lattice strain drives defect formation [47]. In comparison, the presence of the CdSe_x_S_1-x_ interfacial alloy in CdSe/CdS distributes the interfacial stresses, thus providing the opportunity to grow large shells without inducing additional interfacial stress [52]. Importantly, this enables such materials to exhibit more homogeneous shape and size distributions compared to their lattice strained counterparts, as well as, facilitates for high quality core/shell materials to be grown with more complex graded structures, e.g., CdSe/CdS/Cd_0.5_Zn_0.5_S/ZnS. The formation of an alloy layer at the interface between the core and shell materials has been also confirmed for ZnTe/ZnSe dots using the X-ray photoemission spectroscopy (XPS) technique [68]. The comparison between the effective core diameters from TEM and XPS analyses suggested the formation of an alloyed region of around 0.45 nm (i.e., below 2 ML) due to interdiffusion of Te and Se atoms during the dot overgrowth.

One of the most interesting core/shell structures grown through SILAR are the “*giant*” CdSe/CdS QDs, because they possess a shell thickness of up to 20 CdS MLs (with size as large as about 15 nm or more) [44]. The large CdS shell ensures that in this system trapping of electrons (or holes) at the external surface is strongly reduced due to its large distance from the core. Additionally, the spontaneous formation of a CdSe_x_S_1-x_ alloy [69] at the core/shell interface has a twofold beneficial effect: (i) it reduces the tendency to form defect traps due to lattice mismatch and (ii) it smooths the potential barrier experienced by the carriers thus making non-radiative exciton decay less detrimental (*vide infra*).

Despite these factors, the main drawback of “*giant*” CdSe/CdS structures for solution-processable photonic devices are associated with their high volumes, which inherently limit colloidal stability as well as their final density when deposited into a film. Moreover, it has been shown by Javaux et al. [70] that, when operating at room temperature (RT), CdS provides a limited barrier for electron migration towards surface traps even in *giant* QDs. This stems from the fact that the energy difference between CdSe and CdS CBs approaches 0 eV at RT, which allows charge delocalization and increases trapping at the surface.

**Figure 4:**
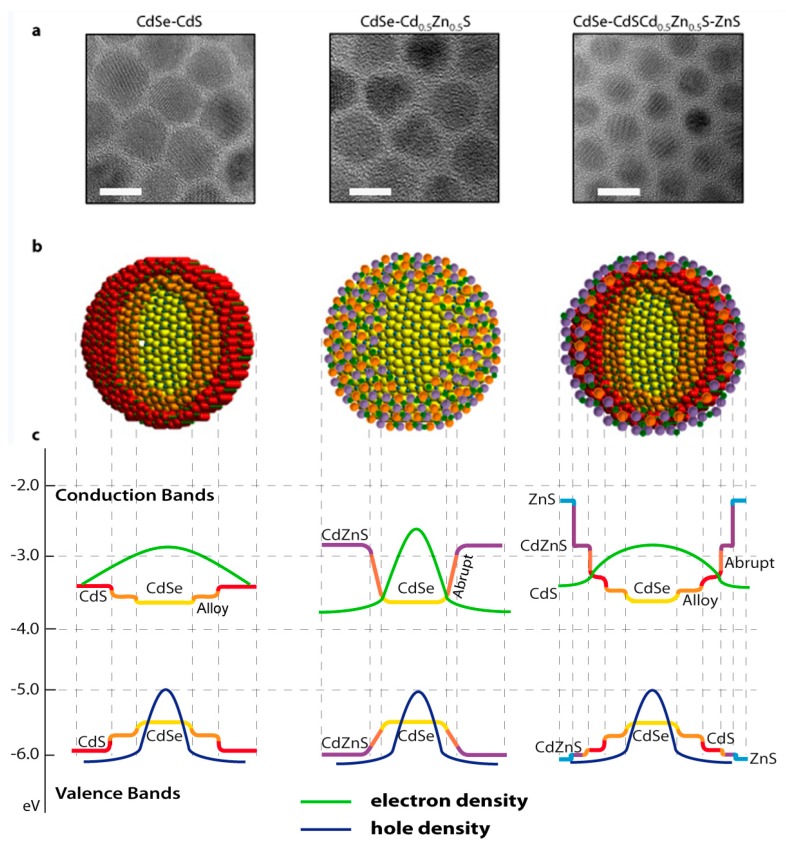
High-resolution transmission electron micrographs (**a**), cross-sectional core-shell structure depiction (**b**) and a schematic representation of the electronic (hole) density distribution (**c**) of CdSe-CdS, CdSe-Cd_0.5_Zn_0.5_S and CdSe-CdS-Cd_0.5_Zn_0.5_S-ZnS QDs. The scale bar in (**a**) represents a 10 nm length. The vertical scale bar in (**c**) is relative to vacuum and the represented energy levels are that of the bulk materials. (**b**) is reprinted with permission from reference [66]. Copyright 2013 American Chemical Society.

Following Javaux’s considerations, in order to keep the QD dimensions small, while maintaining high optical properties, the QD structure should provide a smoothed confinement potential that gradually enhances the electronic barrier to prevent carrier migration towards the external surface. The easiest way to obtain such a configuration is to grow a QD with a *graded* shell composition that possesses a radial increase of the potential barrier from the core to the outer shell. CdSe/CdS/Cd_0.5_Zn_0.5_S/ZnS QDs are an example of a system that is not only synthetically readily accessible using SILAR [70], but overcomes all of the inherent disadvantages presented in pure CdSe/CdS QDs: (i) the inherent alloying between CdSe and CdS minimizes interfacial defect and smooths the confinement potential [67]; (ii) the sequentially higher band gaps provided by Cd_0.5_Zn_0.5_S and ZnS overlayers provide a gradually confining electronic barrier for both electrons and holes; (iii) the ZnS layer aids to provide enhanced photo-oxidation resistance; and, above all; (iv) all these requirements can be readily achieved within a compact hetero-structure containing less than 6 ML of shelling material (Figure 4).

## 4. Fighting Auger Recombination

In order to harness the favorable luminescence and optical gain properties of QDs, including those of core/shell structures, two major non-radiative recombination pathways inherent in such systems must first be minimized: trapping of carriers at defects and *Auger recombination* of excitons [71].

**Figure 5:**
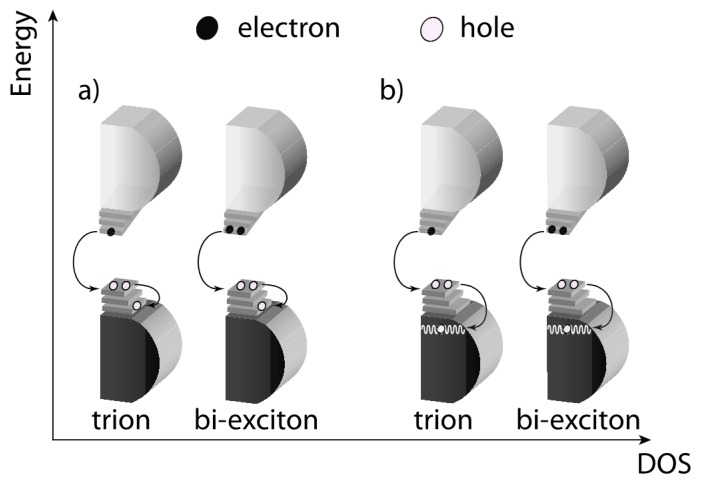
Schematic diagram of non-radiative AR in a QD that acquires a positive charge: in both trion and biexciton case the exciton energy can ejected the extra hole towards discrete levels (**a**) or into the continuum (**b**).

AR is a three-particle phenomenon and occurs when an exciton recombines in a non-radiative way by transferring its energy to a nearby electron (hole) [11]. The electron (hole) is either promoted to a higher level (Figure 5a, with reference to hole excitation) or ionized into the band continuum (Figure 5b). In QDs such a process is more probable either in the presence of: (i) more than one exciton per dot (Figure 5, right schemes) or; (ii) one exciton in a QD containing an excess charge, i.e., a so-called *trion* (Figure 5, left schemes). Long-lived excess charges responsible for Auger non-radiative recombination are mostly localized on the QD surface [71,72].

In the literature two main strategies have been pursued to increase the light emission properties of QDs: (i) localization of excitons away from the outer surface (where traps are mainly localized) and (ii) smoothing of the confinement potential, that allows reduction by three orders of magnitude for the Auger recombination rate [73].

Direct control of Coulomb interactions between electrons and holes within QDs and with charged traps, respectively, is the main way to manage AR. This can be accomplished through wavefunction engineering using structurally controlled core/shell heterostructures. In the case of emission from single excitons a high degree of overlap between *e* and *h* wavefunctions should be maintained. A direct outcome of this strong overlap is an enhanced Coulomb interaction between the carriers that favors fast radiative decay and high QY. Increasing the volume of a core-only nanocrystal reduces this inter-carrier Coulomb interaction and makes trapping at surface defects the most dominant non-radiative process. In this case, it is the Coulomb interaction with charged defects that reduces the luminescence QY by way of non-radiative AR. For the case of multiply excited QDs, a condition that is required to observe optical gain in such systems (*vide infra*), it is the Coulomb interaction between charge carriers of multiple excitons that triggers the AR.

In addition to the strategies to reduce Auger non-radiative recombination, much effort has also been spent on optimizing the surface properties of QDs and reducing the local density of charged defects through organic ligand selection [6,14,15,46] or through the growth of epitaxial Type-I core/shell structures [4,31]. Organic molecules, sometimes also used for the QD solubilization, have to be accurately selected, since some organic groups can act as additional surface trap state, causing a reduction of the nanocrystal QY. The growth of a wider bandgap inorganic shell on the inner core overcomes this problem, resulting in a significant improvement of the photoluminescence efficiency, while simultaneously reducing the photodegradation and increasing the shelf life. However, in a core/shell QD the high photoluminescence QY is a trade-off between protecting against trapping at the surface defects and causing the introduction of additional traps at the core-shell interface, often influenced by the lattice mismatch between different materials [74]. The control of defects is still challenging and a QD ensemble, used in most practical applications, is inherently poly-dispersed because of dot-to-dot variability in size, surface and core-shell interface properties. It is indeed demonstrated that the shell coverage is sometimes inhomogeneous (i.e., with incomplete, “pinched” or “divot” shapes) and inefficient from dot to dot [75].

A successful route to high QY QDs, based on the simple and effective idea of using large shells, was independently demonstrated in 2008 by Klimov [52] and by Dubertret [76]. These so-called “*giant*” *QDs* consist of a core embedded within a thick shell that effectively confines the carriers in the core (Type-I structure) and hinders their tunneling or diffusion towards the surface [69,73]. In addition, in CdSe/CdS QDs the reduction of non-radiative AR benefits from the formation of a CdSe_x_S_1-x_ alloy layer at the core-shell interface. Such layer has the effect of smoothing the interface potential barrier. In a nutshell, the rate of AR is large if the wavefunction overlap between the initial carriers and the final hot carrier is also large. For the carriers of the exciton, this may occur close to the core/shell interfaces if the associated confinement potential is abrupt. Smoothing of this potential acts to reduce this degree of overlap, thereby providing a facile approach to reducing the AR rate by up to one order of magnitude. Exploiting a similar concept, nanocrystals having quasi Type-II or Type-II electronic structure have been studied. Research on colloidal Type-II systems was triggered by the seminal work of Bawendi et al., describing the synthesis and optical properties of CdTe/CdSe and CdSe/ZnTe nanocrystals [77]. Type-II QDs are characterized by a partial or complete separation of the electron and the hole wavefunctions. When multiple excitons are created in the QD, the spatial separation of *e* and *h* wavefunctions induces a repulsive exciton-exciton Coulomb interaction, which promotes the reduction of Auger recombination [34,78,79].

Since the AR time is generally described by a volume scaling dependence, a reduction of Auger recombination has been found for elongated shape nanocrystals, such as nanorods [80], nanobundles [81] and nanoplatelets [37]. In addition, also for spherical dots, theoretical calculations on Auger recombination rates in highly confined structures showed that there are some “magic sizes” that promote the almost complete Auger suppression, due to the destructive interference between the initial and final states. Since such AR rates minima have very narrow widths (1–2 Å), random temperature and electric fields fluctuations and size distribution strongly limit the detection of these “magically sized” dots, at least at room temperature [73]. In the future, the development of synthetic procedures for the precise control on the dot size could allow the production of nanostructures with enhanced optical properties in terms of emission yield and optical gain efficiency.

### Influence of Shell Composition on QDs Optical Properties

**Figure 6:**
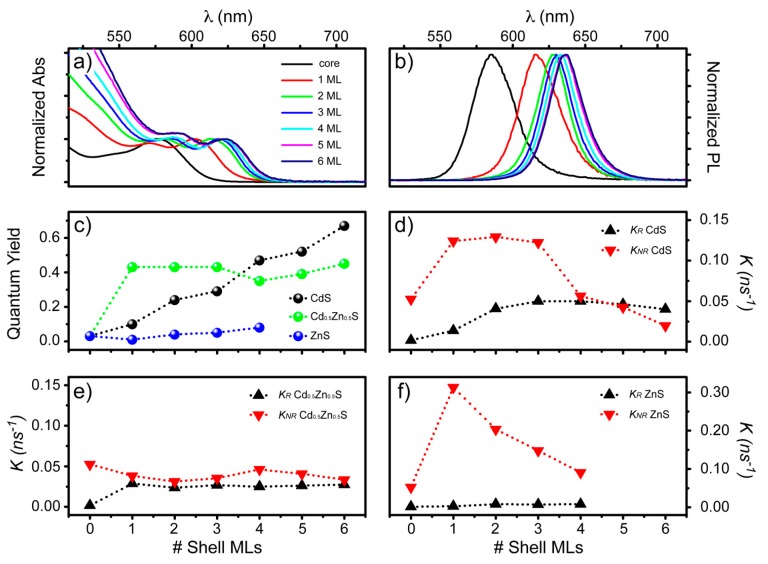
Absorption (**a**) and emission (**b**) spectra of CdSe-CdS QDs as a function of shell ML number; (**c**) QY of core-shell QDs based on CdSe core and different shell as a function of shell ML number; (**d**–**f**) Radiative (k_R_) and non-radiative (k_NR_) recombination rate of CdSe based core/shell as a function of shell type and ML number.

An alternative approach to giant core-shell QDs is to surround the core with a multilayer shell with a varying composition of each monolayer. To clarify why graded core-shell CdSe-CdS-Cd_0.5_Zn_0.5_S-ZnS are highly performing QDs for optical applications [70,82], the correlation of CdSe-Cd_x_Zn_1-x_S QDs emitting properties as a function of shell thickness, structure and composition was recently investigated by our group [83]. Typical CdSe-CdS, absorption (and emission) spectra, with increasing number of shell MLs, are reported in Figure 6a,b. The red-shift of the first excitonic peak (and of the emission peak) that is observed with shell growth is due to the aforementioned delocalization of electrons into the surrounding shell [51,67]. This behavior is observed in all CdSe-Cd_x_Zn_1-x_S QDs, but it is naturally less pronounced in “Zn-rich” materials, i.e., Cd_0.5_Zn_0.5_S and ZnS shell, due to their larger confinement potentials [83].

The QY also exhibits a strong dependence on shell thickness and composition (Figure 6c). Notice that the direct growth of a ZnS shell onto the CdSe cores does not provide any improvement in QY. The lattice mismatch is too large as the synthesis temperature is too low to permit ion interdiffusion [84] and strain is released by defect formation [47,51]. For the intermediate CdSe-Cd_0.5_Zn_0.5_S series, the QY increases from 10% to 45% after the first shell ML deposition and then saturates. Such an abrupt increase is justified with the higher confining potential of Cd_0.5_Zn_0.5_S.

In order to understand the influence of core/shell interfaces and shell thickness on exciton recombination dynamics, time-resolved emission experiments from each CdSe-Cd_x_Zn_1-x_S series were performed. Charge trapping and de-trapping at core/shell and external QD surfaces occurs on the same time scale as exciton recombination. As such, CdSe-Cd_x_Zn_1-x_S QDs exhibit convoluted multiexponential emission decay behavior that can only be interpreted according to a recently derived kinetic model by Jones et al. [85]. This kinetic model considers the physically realistic QD structure and the associated radial traps states, which unequivocally enables the extraction of the radiative (*k_R_*) and non-radiative (*k_NR_*) decay and the trapping/de-trapping rates for different core/shell QDs.

From the results of our systematic analysis, it is evident why graded QDs are high performing materials for light emission applications [70]: (i) they retain in a single entity the high radiative decay rates of CdS; (ii) they exhibit high de-trapping and low non-radiative decay rates of Cd_0.5_Zn_0.5_S; and (iii) they possess the superior confinement potential of ZnS.

## 5. Use of QDs for Light Emission Applications

In the previous sections, we focused the attention on the nanoscale properties of semiconductor QDs in terms of surface chemistry, interface features at the core-shell region, size and shape properties. All these characteristics can affect their electronic and optical properties. In the following chapter, we will summarize the foremost results of two applications of QDs as light emitters: the optical gain and the displays applications.

### 5.1. Optical Gain and Lasing Applications

Colloidal nanocrystals have been extensively studied as prospective materials for the realization of solution-processed lasing media with broadly tunable emission wavelengths. Optical gain, defined in terms of the difference between the stimulated emission and absorption rates, is the main property to be boosted to reach high performances for lasing applications.

Both theoretical and experimental data have clearly demonstrated that optical-gain performances improve from the suppression of Auger recombination [83,86,87]. Population inversion in nanocrystal takes place when the average number of excitons per nanocrystal is greater than 1, this means that the optical amplification is due to multiexcitonic excitation [88]. The Amplified Spontaneous Emission (ASE) originates from the relaxation between the biexcitonic and the single exciton states. Fast relaxation of optical gain resulting from Auger non-radiative decay represents a major complication for both generating population inversion and maintaining it for time durations sufficient for the development of the lasing regime; longer optical gain lifetimes support inversion buildup, responsible for lasing action, and yield lower lasing thresholds. It has been also demonstrated that it is possible to obtain optical gain also in the single-exciton regime using Type-II core/shell CdS/ZnSe hetero-nanocrystals [87]. The resulting imbalance between negative and positive charges following a charge separation process produces a strong local electric field and a transient Stark shift of the absorption spectrum. This eliminates absorption losses at the nanocrystal emission wavelength, allowing optical gain using single-exciton states.

Giant QDs [89], consisting of a small CdSe core overcoated with a thick shell of up to 19 CdS monolayers, and CdSe/ZnS core/shell colloidal quantum rods, with 1.5–2 ZnS shell layers, present a significant suppression of Auger recombination. The lowest ASE threshold (6 μJ/cm^2^) and the highest gain coefficient (521 cm^−1^) have been measured for CdSe colloidal Quantum wells under fs pumping, thanks to their large oscillator strength and consequently large spontaneous and stimulated cross sections. Importantly, these features have also allowed the activation of stimulated emission under CW excitation at 444 nm with a threshold of 6.5 W/cm^2^ [90]. Graded QDs and nanorods [91] possess slightly reduced ASE characteristics, but are none-the-less more than sufficient to achieve lasing within different prototype structures (see Table 1).

To fabricate lasers, NCs are incorporated within an appropriate micro-cavity to provide optical feedback [25,92,93,94,95,96] or dispersed in solution in an open access microcavity [97]. Devices without cavity have also been investigated based on the random laser action [98] and pioneering experiments have shown that also a single QD emitter can drive a nanocavity system into stimulated emission [99,100]. Lasing devices, with high quality performances, have been experimentally tested by using different micro-cavities, like micro-ring [25], spherical resonators [92], distributed Bragg reflectors (DBR) [93,97], and distributed feedback (DFB) [94]. Some laser prototypes have also been demonstrated to operate both under direct one-photon optical pumping and through a two-photon absorption induced up-conversion mechanism, i.e., producing a laser beam with wavelength in the visible range upon IR excitation [95,96].

Two-photon excitation is achieved through the simultaneous absorption of two photons, which induces an electronic transition from the ground to an excited state via virtual states [101,102]. It possesses several unique features such as higher spatial resolution and longer penetration depth when operating in the semitransparent infrared window of biological media. Additionally, for the generation and wavelength tuning of coherent light, the absence of a phase matching requirement in the two-photon emission process, makes it highly attractive over other nonlinear frequency conversion techniques (e.g., optical harmonic generation), as this permits its application to a much wider range of resonator designs and gain media. Given the advent of robust, low-cost, versatile, and compact IR laser sources (e.g., fiber lasers), two-photon excitation has become a viable technique for the generation of coherent light via the attainment of optical gain and lasing in colloidal semiconductor NCs [103,104,105]. Moreover, in contrast to UV-vis excitation, optical pumping via two-photon excitation at IR wavelengths has another advantage—it can reduce the adverse photodamage process on photoactive materials.

**Figure 7:**
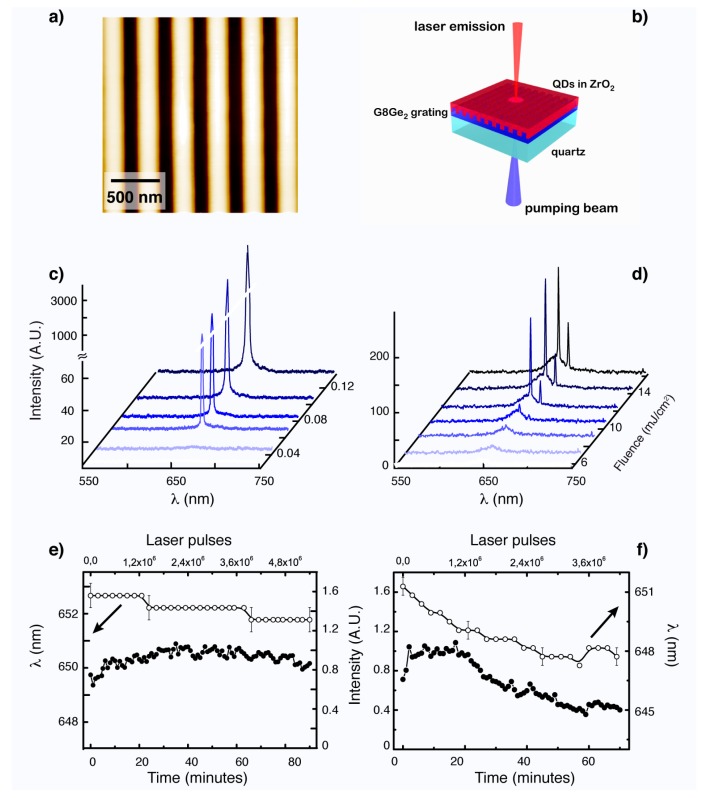
DFB grating (**a**) and scheme of the laser prototype (**b**). Laser emission measurements, carried out by one- (**c**) and two-photon (**d**) optical pumping, show lasing emission above the optical gain threshold: TM (with electric field polarized perpendicular to the grating grooves) and TE (with polarization in the plane of incidence parallel to the grating grooves) lasing modes are detected. The threshold fluences are 0.077 ± 0.023 and 0.108 ± 0.056 mJ/cm^2^ for the one-photon pumped TM and TE modes respectively, and 8.3 ± 2.5 and 11.4 ± 3.2 mJ/cm^2^ for the up-converted ones. Photostability of TM mode at one- (**e**) and two-photon pumping (**f**). Reprinted with permission from reference [95]. Copyright 2011 The Royal Society of Chemistry.

As previously described, graded core/shell QDs combine favorable optical properties, like high absorption cross-sections and QY, extremely high photostability under laser irradiation, high optical gain efficiency and low ASE thresholds to be employed for developing QD based micro-lasers [82,95]. They are also particularly interesting candidates as active material for miniaturized lasers since their structure can be precisely controlled and their surface chemistry can be readily modified, which allows for their efficient inclusion into solid films. Our group has recently shown that graded QDs hosted in a suitable ZrO_2_ sol-gel matrix are photo-chemically stable and can be prepared at sufficiently low temperatures to retain the beneficial optical properties of the NCs [103,106]. Sol-gel techniques, in general, enable the preparation of solution processable layers with tailored optical and mechanical properties, such as controlled thickness, suitable refractive index and stiffness [107]. These properties have been harnessed to fabricate a laser device prototype by simply depositing graded QDs embedded within a ZrO_2_ host on top of a 1D DFB grating, which was obtained through nano-imprinting of a germania-based sol-gel matrix (Figure 7a,b) [95]. The combined effect of active film thickness, refractive index and grating period, entails direct tunability of the laser’s output spectra within the gain profile [108]. These features have enabled single mode lasers (Figure 7c,d) to be demonstrated with a low lasing threshold of 77 μJ/cm^2^ (Figure 7c,d). Such lasers can operate also in an up-converted configuration at 800 nm excitation, giving lasing thresholds two orders of magnitude lower than those of organic small molecules [109] and about one order of magnitude smaller than those of QDs micro-bead systems [92] and CdSe/CdS nanoplatelets [93].

**Figure 8:**
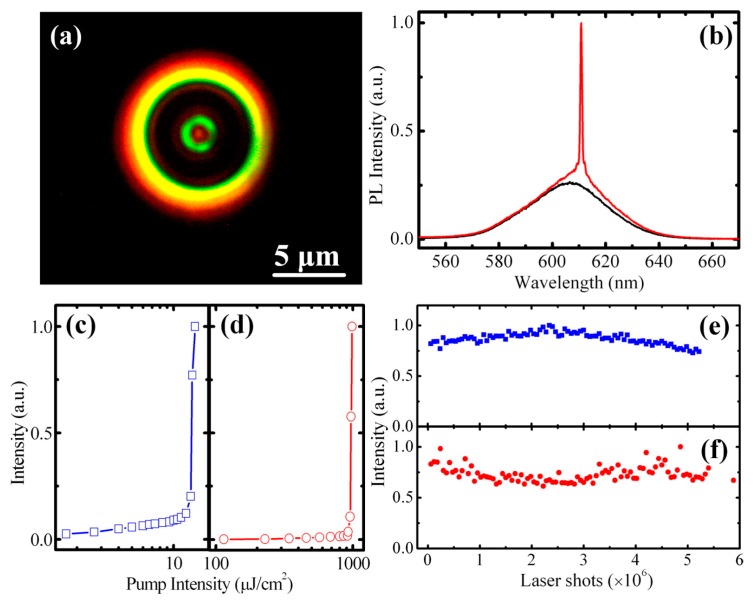
(**a**) Optical image of a 5 μm silica microsphere coated with a CdSe/CdS NR film; (**b**) Emission spectra of NR coated microsphere below (800 μJ/cm^2^) and above (900 μJ/cm^2^) lasing threshold under one-photon pumping; (**c**,**d**) Emission intensity as a function of pump intensity for one- (blue) and two-photon excitation (red). Photo-stability under one- (**e**) and two-photon (**f**) excitation. Reprinted with permission from reference [110]. Copyright 2012 American Chemical Society.

Lasing under one- and two-photon pumping has been obtained also using seeded CdSe/CdS nanorod (NR) heterostructures, comprising as the optical gain media a spherical CdSe core of 2.4 nm diameter, encapsulated by a rod-like CdS shell of 39 nm [110]. While the CdS shell functions as an antenna in light harvesting, emission from the rod originates primarily from the CdSe core. One of the main advantages of this optical configuration is the realization of a material with variable emission wavelength: emission can be tuned by adjusting the physical dimensions of the rod-like shell and the spherical core. For 39 nm long NRs, a two-photon absorption cross-section value of 2.3 × 10^5^ GM (where 1 GM = 10^−50^ cm^4^·s/photon) was measured [110], which is 2 to 4 orders of magnitude larger than that previously reported for spherical semiconductor QDs and about 4 orders of magnitude larger than that of typical organic dyes [102]. Importantly, two-photon absorption cross-section can be varied independently of the CdSe core dimension, whose size primarily determines the emission wavelength. The advantages of size-dependent emission afforded by the quantum- confined CdSe core are therefore preserved in these heterostructures. Figure 8a shows an optical image of a typical NR-coated microsphere under optical excitation [110]. These nanostructures have been characterized by room temperature ASE, via two-photon excitation at 800 nm, showing extremely low thresholds (1.5 mJ/cm^2^). NRs have also been used as gain medium for two-photon pumped lasing using a spherical optical cavity. To this end, CdSe/CdS NRs were chemically functionalized and incorporated into a sol-gel silica matrix to permit the adhesion of a thin layer of NRs-silica onto the exterior of commercially available silica microspheres (of 5.0 μm diameter) and to increase the damage resistance of samples to prolonged pulsed excitation. Under two-photon pumping a single-mode lasing was achieved (Figure 8b). It is characterized by a threshold fluence of 990 μJ/cm^2^, a laser line with a full-width-at-half-maximum (FWHM) of 0.75 nm, corresponding to a Q-factor of 800 and a photostability for over 6 × 10^6^ laser shots under ambient conditions, evaluated by monitoring the lasing intensity as a function of time under a continuous irradiation at a 1 kHz repetition rate under ambient conditions.

Among Nanoplatelets, CdSe/CdS core/crown NPLs (or also denoted as CdSe/CdS nano-heteroplatelets with CdS wings) are highly attractive due to their photophysical properties [93]. The peak emission wavelength of the core/crown NPLs does not change (except for a few nanometers red shift) as compared to their core-only counterparts owing to the confinement of the excitons in the core. The core/crown NPLs preserve the narrow spontaneous emission properties of the core-only NPLs, while the absorption cross section of the NPL is boosted due to the energy transfer from the crown to the core. Finally, with the growth of CdS crown, the quantum yield and stability of the NPLs are enhanced. Optical performances of a core only sample, constituted by four CdSe monolayers with a final shape varying between square and rectangle and an average long-edge length of 16.8 nm, have been compared to the optical performances of core/crown NPLs, with a CdS crown lateral growth and an average size of 25 nm. The core/crown NPLs demonstrate substantially lowered ASE thresholds as compared to the core-only NPLs: the one- and two-photon pumped ASE thresholds have been determined to be 41 μJ/cm^2^ and 4.5 mJ/cm^2^, respectively, for the core/crown sample, while 214 μJ/cm^2^ and 8.2 mJ/cm^2^, respectively, for the core only sample. Besides, the gain coefficient of the core/crown NPLs is measured as high as 650 cm^−1^.

**Figure 9:**
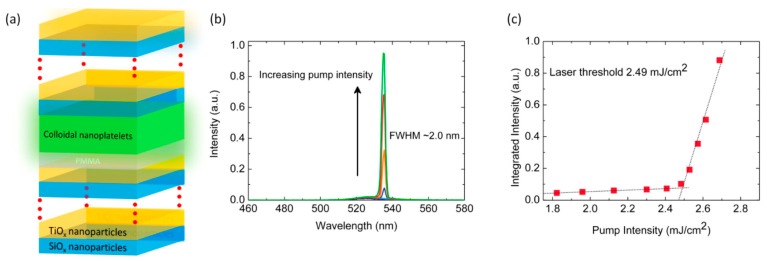
(**a**) Scheme of the VCSEL of the NPLs employ DBR with a six bilayer stack of SiO_2_ and TiO_2_ nanoparticles each; (**b**) Emission spectrum of VCSEL at increasing pumping intensity; (**c**) Integrated emission intensity vs. 2-photon pump intensity. Reprinted with permission from reference [93]. Copyright 2014 American Chemical Society.

Lasing with core/crown NPLs has been experimented in a Distributed Bragg Reflectors configuration, made out of alternatively stacked SiO_2_ and TiO_2_ nanoparticles. Employing six bilayers of the SiO_2_/TiO_2_, a DBR system with peak reflectively as high as 91.93% has been obtained. The vertical cavity surface-emitting laser (VCSEL) of the NPLs has been realized by sandwiching 25 nm size core/crown NPLs between two DBRs (Figure 9). The figure shows the emission spectra of VCSELs under two-photon pumping as the pump intensity is progressively increased: above the lasing threshold (2.49 mJ/cm^2^) peak at 535 nm emerges with a FWHM of 2 nm, corresponding to a Q-factor of 270 [93].

**Figure 10:**
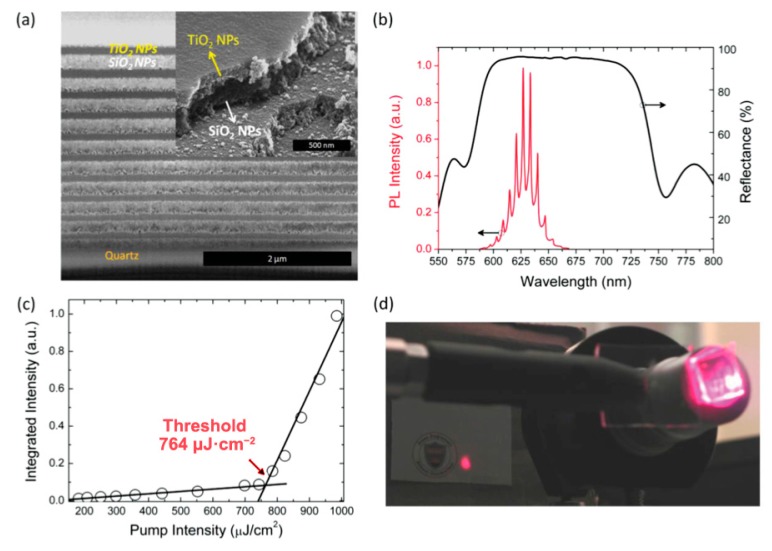
(**a**) Scanning electron microscopy cross-sectional image of DBR structure; (**b**) Surface normal reflectance of DBR with ten-bilayer of TiO_2_ and SiO_2_ nanoparticles along with the emission of the QDs in the cavity with 15 μm optical thickness; (**c**) Emission intensity vs. pump intensity for frequency up-converted VCSEL; (**d**) Photographic image of the lasing spot from the VCSEL. Reprinted with permission from reference [111]. Copyright 2015 Wiley.

Recently, very high performance and extremely stable optical gain media, via both one- and two-photon pumping, have been obtained using tailored CdSe-core/CdS-shell QDs [111]. High-quality zinc-blende (ZB) CdSe nanocrystals have been grown followed by slow CdS-shell growth at high temperature to improve the crystal quality. By growing the CdS shell at an exceptionally high temperature (310 °C) and at a very slow rate (6 monolayers in 3 h), a smooth Cd(Se,S) interface layer with a thickness of 2–4 monolayers is likely to have been created. This relatively thin CdS shell helps to combine the small nanocrystal size, high photoluminescence quantum yield and the high performance optical gain—a low threshold of 29 μJ/cm^2^ and extremely high stability (1.8 × 10^7^ laser shots under one-photon pumping). The high-performance QD gain medium is further demonstrated in an all-colloidal vertical-cavity surface-emitting laser (AC–VCSEL) using simple and solution-processed DBR of just 96% reflectivity. With the solution-processed DBRs and the tailored core/shell QDs, an ultralow frequency up-converted lasing threshold of 764 μJ·cm^−^^2^ have been achieved (Figure 10) [111].

The use of such engineered nanocrystals (graded QDs, NRs or NPLs), possessing lower one- and two-photon pumped lasing thresholds than the spherical semiconductor QDs, allows the implementation of all-solution-processed QD-laser technologies, representing the leading most cost-effective approach towards high-performance full-color lasers with single material technology.

### 5.2. Display Applications

The efficiency and color quality of a liquid crystal display (LCD) is mainly dictated by the quality of the white backlight module and the optimal monochromaticity of filters used for the selection of single Red-Green-Blue subpixel components. To this end, large efforts have been devoted to the generation of new strategies to meet the full color gamut accordingly to the National Television Systems Committee (NTSC) standard requirements (CIE 1931 [112] or CIE 1976 [113]). In addition to such fundamental color and brightness requirements, other features are important for the optimal performing display, such as the fast response time, wide viewing angle, black background (in particular in a dark place), low energy consumption and costs, flexibility and compactness. Up to few years ago, the organic light emitting diode (OLED) technology has shown the best performances in terms of luminosity, wide gamut range, response time, true black light and compactness. However, the high production costs and commercial scaling issues limit the widespread use of OLED to smaller devices [114].

Recently, inorganic QDs are emerging as one of the most promising emitting materials for next generation LCDs. Thanks to their intrinsic optical properties, such as their broad absorption band, narrow emission spectra, high fluorescence quantum yield, high photostability and controllable emission and surface properties, QDs based LED is now showing performances comparable or even better than OLED devices [2,3,115]. For display applications, QDs can be used either exploiting their photoluminescence for LCD backlight unit [116,117] or their electroluminescence for QDs-light emitting diodes (QLED) [118,119].

**Figure 11:**
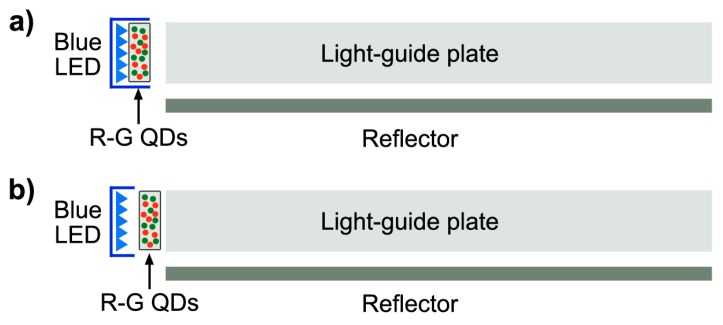
Depiction of the on-edge implementation QLED geometries. QDs are placed within the Blue LED package, which is coupled to the light guide (**a**) or between the Blue LED package and the light guide plate (**b**).

One of the technologies to employ QDs as down-conversion emitters involves the encapsulation of green and red QDs in a tube or “rail”, placed directly in front of blue LEDs on the edge of the panel (see Figure 11). By combining the blue optical excitation with the QDs emission in the red and green regions, all three R-G-B primaries are produced. From a technological point of view, there are two different packaging strategies for the preparation of QLED, named respectively *remote-type* and *on-chip type*. The main difference is that in the first case the QDs are included into a flat polymeric slab, which is further used to cover the Blue-LED chip, while in the second type the QDs-polymeric mixture is solidified directly on top of the LED chip. In both cases, the QD-LED assembly needs a further encapsulation for the protection towards oxygen, moisture and temperature. One of the most used hosting polymers is PMMA [120,121]. The final structure of the protective coating can also influence the overall light output efficiency. Throughout all the processing steps, there are many critical aspects that need to be further optimized in order to increase the light emission performances. They include: the surface chemistry properties at the interface between the QDs and polymeric matrix to avoid agglomeration and reduction of QD emission efficiency [122,123], the right mixture of different QDs components to achieve high-color rendering ability [124,125,126] and the encapsulation matrix able to protect the chip and to prolong its lifetime [127,128]. For an overview on the main strategies reported in literature, in particular for highly efficient white-light LED, refer to the recent review written by Xie and colleagues [129].

The in-rail approach has already commercially demonstrated within specific Sony displays; however the additional mechanical element added to the LCD configuration does place restrictions on the possibility of converting it into a more light and flexible display [130,131].

**Figure 12:**
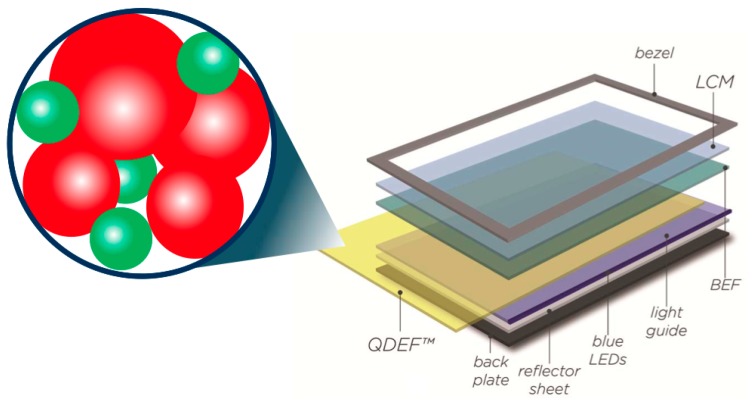
QDEF integration into an LCD backlight. QDEF is sandwiched between the LCM (Liquid Crystal Module), the BEF (Brightness Enhancement Film) and the blue LED. Reprinted with permission from Reference [133]. Copyright 2012, SID DIGEST.

Two alternative approaches have been proposed respectively by NanoSys^®^ in collaboration with 3M^®^ and by QD Vision^®^ [130,132,133]. NanoSys proposed a Quantum Dot Enhancement Film (QDEF), a flat film doped with Red and Green QDs placed in front of the backlight blue LED illumination within the LCD architecture (see Figure 12).

**Figure 13:**
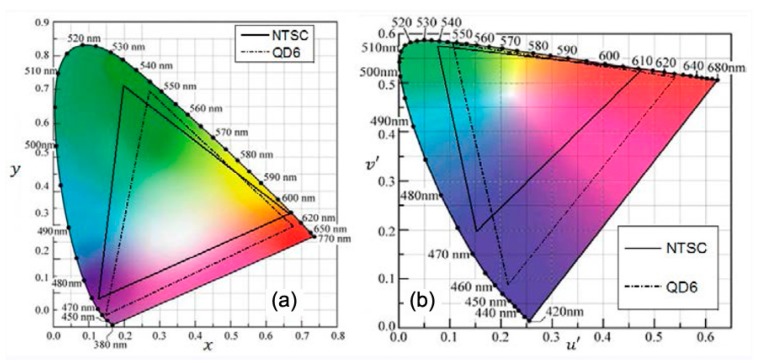
(**a**) Color primaries of a QDs sample (named QD6) as well as NTSC standard in CIE 1931 color space; and (**b**) Color primaries of QD 6 and NTSC standard in CIE 1976 color space. Reprinted with permission from Reference [117]. Copyright 2013, OSA.

In this geometry, the LED excitation is spread over the entire area, which enables all three colors to produce a white light back-illumination across the panel with high quality, uniformity and increased device lifetime. However, the QDs have to be appropriately functionalized for the efficient inclusion in polymeric matrices and the QDEF has to be sandwiched between two layers for preventing the moisture and oxygen that could adversely affect the QDs emission efficiency. The resulting device exceeds the color output and efficiencies of OLED, producing 50% more colors within the NTSC 1953 color gamut and with emission properties invariable over 30,000 working hours, even if the production cost is still high [134]. Following the same strategy, Luo and colleagues demonstrated the possibility of increasing the color gamut over 120% NTSC in CIE 1931 color space and 140% NTSC in CIE 1976 color space, as depicted in Figure 13, using CdS_x_Se_1-x_/ZnS core-shell QDs from Cytodiagnostics. The particle size was between 5.5 nm and 6.5 nm to modify the emission wavelength [117]. These results exceed the data obtained for the displays produced by Samsung [116] and Nanosys [133], covering respectively 104.3% and 109% NTSC in CIE 1931 color space.

Instead of using a flat and large film doped with QDs, QD Vision developed a new product having the QDs integrated in a LED array placed in the panel edge. This allows a reduction to 1% of QDs with respect to the amount used in QDEF film. In parallel, they are also working for replacing organic material in OLED with inorganic QDs for direct light generation with better definition of R-G-B emission wavelength. In this case, by suspending QDs in appropriate solvents and polymeric matrices, it will be possible to develop innovative processes based on direct printing maintaining higher emission efficiencies with respect to organic molecules [132].

Inorganic QDs have also been largely studied as emitting materials in electrically pumped light emitting devices (QLED). Many works have explored the possibility of producing single color QLEDs, reaching the best performances with II-IV group visible emitting QDs [135]. However, QLEDs still exhibit low efficiency if compared to other inorganic or organic LED. This is mainly due to the misalignment between the QDs energy levels and the levels of the coupled charge transport layers. Indeed, the misalignment with the hole transport layer (HTL) prevents the hole injection between the QDs and the HTL, favoring the adverse effect of exciton quenching. Many approaches have been proposed, including the use of a sensitizer that transfers the excitation energy to the QDs via Förster resonance energy transfer [136] or the use of an inorganic electron transport layer (ETL) and an organic HTL. With the latter configuration, the highest external quantum efficiencies are found to be 10.7%, 14.5%, 20.5% for blue, green, red QLED respectively [135,137]. Stability remains a well-known issue for the blue and green QLEDs.

**Figure 14:**
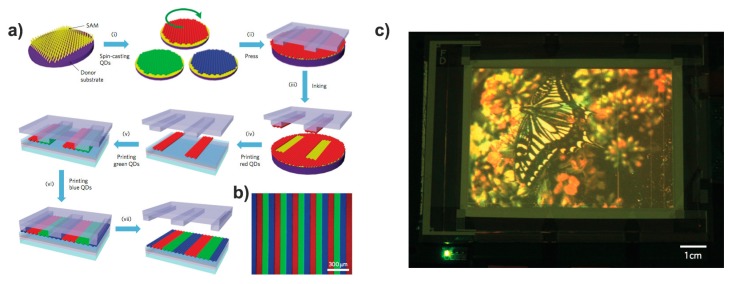
(**a**) Transfer printing process for micropatterning of quantum dots; (**b**) Fluorescence image of the RGB QD microstripes onto the glass substrate; (**c**) Electroluminescence image of a 4-inch full-color QD display using a HIZO TFT backplane with a 320 × 240 pixel array. Reprinted with permission from Reference [138]. Copyright 2011, Macmillan Publishers Ltd.

Meanwhile, the inclusion of all three primary colors into one device is still posing critical issues regarding the processing of the final device. Various strategies are explored using also different processing techniques. Kim et al. fabricated a full color QDs display using a solvent-free transfer microprinting method where microstripes of single red, green, and blue emitting QDs are used to sequential transfer of QDs to a pixelated display panel (see Figure 14). Thanks to the elastic behavior of PDMS stamp, this printing method can transfer QDs onto flexible substrates, giving exciting opportunities for scaling up the process to a roll-to-roll system [138].

**Figure 15:**
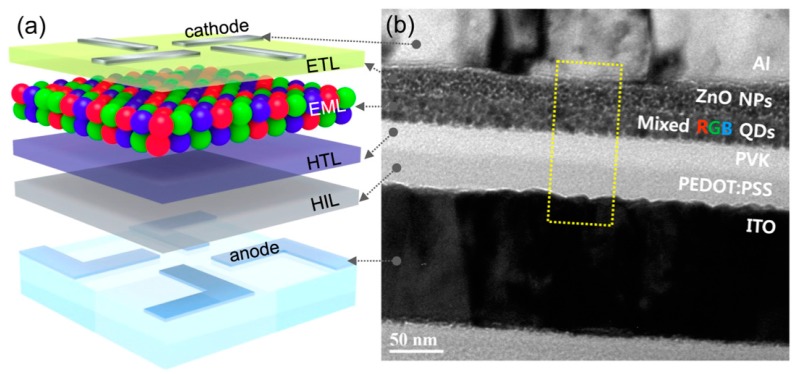
(**a**) Device structure and (**b**) cross-sectional TEM micrograph of all-solution processed full-color QLED. Reprinted with permission from Reference [21]. Copyright 2015, American Chemical Society.

Other deposition processes involve full solution processing architectures, using the spin casting technique for the deposition of single colour QDs in each subpixel [137,139], or the deposition of multiple stacked layers by combining solution/vacuum methods [140]. Recently, Lee at al. demonstrated all-solution-processed fabrication of a highly efficient, bright full-color QLED, where RGB QDs-mixed emitting multilayer is sandwiched with poly(9-vinlycarbazole) and ZnO NPs layer acting respectively as HTL and ETL counterparts (see Figure 15). In Lee’s work high-quality fluorescent blue (CdZnS/ZnS), green (CdZnSeS/ZnS), and red (CdSeS/ZnS) QDs were mixed together, obtaining a color gamut of about 126% relative to CIE standard [21].

Most of the studies on QLED report on the application of quite simple QDs based on a core/shell structure, having a II-VI group core and ZnS as shell. To further improve the device performance, important challenges need to be addressed for a widespread use, such as the proper inclusion of QDs in a shell to prevent excitation or heat damage, new encapsulants must be developed and a better engineering of the energy level alignment across all interfaces to achieve a higher external quantum efficiency. Finally, one of the biggest challenges is related to the use of an eco-friendlier emitting material with respect to Cd-based nanostructures. In this context, some structures based on III-V InP [141] and In_x_Zn_x_P QDs [142] have been studied, although critical issues regarding the complete surface passivation and lattice mismatch at the core/shell interface for such systems still remain under investigation.

## 6. Conclusions and Outlook

In recent years, much progress has been made in understanding the structure and dynamics of semiconductor QDs with a view towards their envisaged applications in photonics, optoelectronics, medical diagnostics and therapy. Smart engineering of NC heterostructures, known as core/shell QDs, has emerged as the most successful strategy for gaining control on detrimental processes such as carrier trapping at defects and non-radiative exciton decay, particularly Auger recombination.

In this review, paper we have provided experimental support to the idea that suitably engineered core/graded-shell QDs, prepared by exerting atom-by-atom control on the growth of monolayers of varying composition, provides the best results for maintaining the exceptionally favorable linear and non-linear optical properties, photoluminescence and optical gain, while keeping the synthesis facile and producing QDs well suited for light emitting applications.

Among the numerous prospective applications of semiconductor NCs in photonic technologies, some of which have already reached the marketplace, solid-state laser emitters can greatly profit from QDs as efficient gain materials. This goal will only be achieved if QDs-doped matrices that preserve their favorable photophysical characteristics and are chemically and photo-chemically stable are realized. Progress towards fabricating low threshold, solution processed DFB lasers that are optically pumped using one- and two-photon absorption processes have already provided proof-of-concept, with ample space for improvement by optimization of components and design.

The use of QDs in display devices is gradually gaining its own market share by competing effectively with the LCD and OLED technologies. The high color purity and solution-processable nature of QDs are particularly appealing for such a market. The exploitation of the exceptional photoluminescence properties of QDs for LCD backlighting has already advanced to commercial levels. The color output and efficiency exceed those of OLED, enabling us to approach the full color gamut according to the standard NTSC requirements. The next big challenge is to develop the electroluminescence properties of QD to a similar state. All the exceptional light emitting properties are retained for QLED devices, which provides them with great perspectives for the development of next generation display technologies. However, further efforts are required to progress in our understanding of energy and charge transfer processes in QD films to ensure higher external efficiencies and long term stabilities. The choice of QDs to be used in QLEDs must consider high photoluminescence in dispersed solutions as a starting criterion, which means low trap densities and reduction of Auger recombination. However, in QD films the role of recombination at surface traps is amplified because energy transfer brings into play the trapping at all the QDs visited by energy migration. The formation of charged QDs (i.e., trions) that amplify the AR, the effect of the electric field applied to bias the device, the efficient coupling with the hole and electron transport layers are other factors that must be taken into account. Here too, the continuous progress in producing semiconductor NCs with different architectures (chiefly core/shell ones) and embodying multiple functionalities is providing exciting opportunities for the fundamental understanding of optical processes at the nanoscale and for engineering nanostructures for application in display and energy technologies.

## Figures and Tables

**Table 1:** Summary of ASE and Lasing parameters for different classes of NCs.

Sample	Gain Coefficient (cm^−1^)	1-Photon ASE Threshold (μJ/cm^2^)	2-Photon ASE Threshold (mJ/cm^2^)	Laser Cavity	Stability (Laser Shots)	1-Photon Lasing Threshold (μJ/cm^2^)	2-Photon Lasing Threshold (mJ/cm^2^)	Q-Factor
**Giant QDs** [89]		26		---				
**Quantum wells** [89,90]	521 (fs-pump) 121 (CW-pump)	6						
**NR** [91]	350 (until 120 K)	2.5 × 10^5^						
**Graded QDs** [82]	180	170	12.8	DFB		77	8.3	650
**CdSe/CdS NR** [110]			1.5	Spherical resonator	6 × 10^6^ (1 & 2-ph pumping)	12	0.99	800
**NPLs 25 nm core/crown** [93]	650	41	4.5	DBR	10^4^–10^5^	---	2.49	270
**CdSe/CdSeS/CdS** [111]	120	29	5.02	DBR	1.8 × 10^7^ (pump int. = 35 μJ/cm^2^)	NO Lasing	0.764	---
